# What Plant Genetic Resources for Food and Agriculture Are Available under the Plant Treaty and Where Is This Information?

**DOI:** 10.3390/plants12233944

**Published:** 2023-11-23

**Authors:** Gaia Gullotta, Johannes M. M. Engels, Michael Halewood

**Affiliations:** Bioversity International, 00153 Rome, Italy; j.engels@cgiar.org (J.M.M.E.); m.halewood@cgiar.org (M.H.)

**Keywords:** Plant Genetic Resources for Food and Agriculture (PGRFA), Multilateral System of Access and Benefit-Sharing (MLS), International Treaty on Plant Genetic Resources for Food and Agriculture (ITPGRFA or Plant Treaty), notification procedures, accessions, *ex situ* conservation, Annex I PGRFA, genebanks

## Abstract

Plant genetic resources for food and agriculture (PGRFA) are the building blocks upon which global food and nutrition security depend and are key to plant breeding for more resistant crops, but how available are they? To understand what PGRFA are available under the mechanisms created by the International Plant Treaty’s access and benefit-sharing, we conducted a comparative analysis of the five largest sources of pooled global data concerning PGRFA, including data conserved by and available to users under the Plant Treaty’s access and benefit-sharing (ABS) mechanism. These data sources were the registry of notification letters maintained by the Plant Treaty Secretariat and four international PGRFA databases: Genesys, European Search Catalogue for Plant Genetic Resources (EURISCO), World Information and Early Warning System on Plant Genetic Resources for Food and Agriculture (WIEWS) and Global Information System on PGRFA (GLIS). Our analysis revealed that a comprehensive and consistent overview of the PGRFA available under the Plant Treaty’s ABS conditions is not available. The GLIS is the most logical longer-term candidate to promote the provision of up-to-date and comprehensive snapshots of what PGRFA the Plant Treaty framework make available, primarily because it provides a mechanism (digital objective identifiers) to link together information from a range of information sources, including Genesys, WIEWS and EUEISCO and other online publications, and data sets concerning PGRFA in the multilateral system. Successful adoption of the GLIS could be promoted by creating novel incentives endorsed by the Governing Body to encourage Contracting Parties, Article 15 organizations, and individuals to share information about the materials they are making available under the Plant Treaty, in addition to the capacity-building for some GLIS users that is also necessary. These incentives could be included among the package of measures currently being considered by the Plant Treaty’s Working Group to Enhance the Functioning of the Multilateral System of Access and Benefit-Sharing.

## 1. Introduction

The International Treaty on Plant Genetic Resources for Food and Agriculture (hereafter called the Plant Treaty) [1] was adopted in 2001 and came into force in 2004. The Plant Treaty established the Multilateral System of Access and Benefit-Sharing (hereinafter referred to as the Multilateral System or MLS), which is designed to facilitate access to the global plant genetic resources for food and agriculture (PGRFA) of 64 crops and forages that are listed in Annex I of the Treaty “for the purposes of research, breeding and training for food and agriculture” [2]. The Multilateral System is also designed to collect and distribute monetary benefits derived from the use of genetic materials in the Multilateral System through the Benefit-Sharing Fund (BSF). The BSF provides financial support through a system of competitive grants to strengthen the capacity of actors in developing countries to conserve and sustainably use PGRFA [3]. In particular, the BSF supports farming communities in developing countries that are facing the challenges of climate change and other threats to food production in improving their food security [3]. The Standing Committee on the Funding Strategy and Resource Mobilization of the Plant Treaty—based on the appraisals and recommendations of the independent Panel of Experts—approves projects to be funded [3]. Recipients who commercialize new PGRFA products derived from materials supplied through the Multilateral System are required to make royalty payments to the BSF if those products are not “available for use without restriction” for further research and breeding. 

Potential users in any Contracting Party country to the Plant Treaty have the right to access plant genetic materials in the Multilateral System without charge or for a fee not exceeding the minimal administrative costs. All transfers of materials under the Multilateral System are subject to the terms of the Standard Material Transfer Agreement (SMTA), which was adopted by the Governing Body of the Plant Treaty in 2006 to regulate the movement of materials [4]. The SMTA constitutes a binding legal agreement between providers and recipients of PGRFA in the Multilateral System. The SMTA sets out, among other things, the permitted uses of transferred materials, benefit-sharing options and payment obligations, dispute resolution procedures and the provider’s obligation to report the transfer to the Governing Body. The SMTA is posted on the Plant Treaty’s website in the six official UN languages, where it is available for download and use by providers and recipients worldwide [4]. Since 2006, more than 100,000 SMTAs have certified the transfer over 6.6 million PGR samples worldwide. Most of the recipients are public sector research organizations in developing countries. Eighty-six percent of those materials were transferred from CGIAR breeding programs and genebanks [5]. 

The Multilateral System includes three categories of Annex I PGRFA, as defined by the source:

- PGRFA that are “under the management and control” of the Contracting Parties (i.e., national governments) and “in the public domain”. These PGRFA are automatically included in the Multilateral System when a Contracting Party ratifies or accedes to the Plant Treaty.
- PGRFA that are voluntarily included by “natural or legal persons”. Since only materials managed and controlled by national governments are automatically included, the Multilateral System depends on additional materials being voluntarily included by national governments (i.e., non-Annex I crops), companies, farmers, private universities, NGOs, provincial governments (in federated states) and others. These entities can voluntarily include materials simply by providing them to someone else using the SMTA (assuming they have the right to do so subject to other potentially applicable national laws) and/or by depositing them in a genebank or other organization that has the mandate to subsequently make these materials available using the SMTA. In some cases, where countries do not have national ABS measures in place (for example, they are not implementing the Nagoya Protocol to the Convention on Biological Diversity) that could also apply to such materials, it may be necessary for the party voluntarily providing the materials to obtain permission from the national competent authority to be able to provide the germplasm.
- PGRFA included in the Multilateral System by international institutions subject to an agreement under Article 15 of the Plant Treaty. To date, 18 international organizations have signed such agreements, including the 11 CGIAR Centers that host international collections and other institutes, such as the Tropical Agricultural Research and Higher Education Center (CATIE), International Center for Biosaline Agriculture (ICBA), International Cocoa Genebank and International Coconut Genebank for the South Pacific [6].

In addition, there are PGRFA that are not included in the Multilateral System since they are not included in the Annex I list but are available to recipients under exactly the same terms and conditions as PGRFA in the Multilateral System when using the SMTA. These non-Annex I materials are also divided into three categories, as defined by the sources. Firstly, some countries, for example, CGN (the Netherlands), IPK (Germany) and NordGen (Nordic countries), make non-Annex I materials held in their national PGRFA collections available in this way. Secondly, in 2007, the Governing Body of the Plant Treaty confirmed that the International Agricultural Research Centers that signed Article 15 agreements could also make non-Annex I PGRFA in their collections available using the SMTA and endorsed the option that an interpretive footnote clarifying that they are being used for non-Annex I materials should be added to those SMTAs [7]. Thirdly, it is possible, albeit not very common, for natural or legal persons to make non-Annex I materials available using the SMTA, if they so want to, subject to national legislation [7]. Henceforth, we will refer to these latter three categories of non-Annex I PGRFA as “PGRFA available under the Plant Treaty framework”.

The Treaty Governing Body has repeatedly invited the Contracting Parties and natural or legal persons to notify the Secretariat about what materials they are making available through the Multilateral System (to be able to actively inform the potential recipients), including during the last session in September 2022 [8]. All such notifications are posted on the Plant Treaty’s website. The notifications received include a wide range of information and details; some refer to the collections, others attach a list of accessions, and others may redirect the reader to a static website or an online database that, over time, is no longer accessible. Given the growing number of Contracting Parties to the Plant Treaty and the difficulty of obtaining useful information from them, the notifications letters are falling out of use. Most new germplasm holders notifying the Treaty about acquired material are adopting the new documentation and notification procedure set in place under the Global Information System for PGRFA (GLIS). 

For potential users, it is essential to know what PGRFA are available under the Plant Treaty framework. It is also critically important for PGRFA-holding institutes to know their rights and obligations regarding the PGRFA they hold. In addition to knowing about the existence of an accession and where it is being conserved, potential users also require related information, which, depending on the user’s objectives, could be information about the material’s origin or its phenotypic or genetic characteristics.

Given the vital importance of such information for breeding, commercialization and development initiatives, and for monitoring the implementation and effectiveness of the Plant Treaty, it is striking that the Plant Treaty does not include an effective legal obligation for Contracting Parties and Article 15 organizations to confirm and publish lists of materials they will make available to recipients under the Plant Treaty framework. 

The Plant Treaty is not entirely silent on this issue. Treaty Article 13.2 states that “Contracting Parties agree to make available information which shall, *inter alia*, encompass catalogues and inventories, information on technologies, results of technical, scientific and socio-economic research, including characterization, evaluation and utilization, regarding those plant genetic resources for food and agriculture under the Multilateral System”. However, the legal weight of this “agreement” is watered-down by the qualification that “such information shall be made available, where non-confidential, subject to applicable law and in accordance with national capabilities”. Further adding to the complexity is the fact that the Treaty states that “such information shall be made available to all Contracting Parties to this Treaty through the information system, provided for in Article 17” (i.e., GLIS). Yet, in 2004, when the Plant Treaty came into force and when the SMTA was adopted in 2006—thereby making the MLS operational—the GLIS had not yet been developed and only became operational in 2017, after the development of a standard procedure to identify material. In addition, there is no language in Article 13.2, or elsewhere in the Treaty or in international institutions’ Article 15 agreements, requiring organizations or national and legal persons to publish lists of the PGRFA they make available under the Plant Treaty framework [8].

It may be that the Treaty negotiators simply assumed that after seven years of negotiations over the terms of access and benefit-sharing, the actual identification of materials available through the Multilateral System was a detail that would take care of itself. Or perhaps it was a deliberate strategy to provide some leeway for Contracting Parties who otherwise would have been reluctant to finalize negotiations if it meant immediately having to confirm, through the publications of lists, all the PGRFA within their borders for which they were now committed to providing facilitated access. As interesting as it is to consider the reasons for this gap in the architecture and logic of the Plant Treaty, it is beyond the scope of this paper. Instead, this paper addresses the following more focused, key question: *What PGRFA are available under the framework of the Plant Treaty and where is this information available?*

Not surprisingly, several initiatives have tried to address this gap in the Plant Treaty’s architecture, creating mechanisms by which Contracting Parties, natural or legal persons and Article 15 organizations can voluntarily—because it is not required by the Plant Treaty—share information about the PGRFA they are making available under the Plant Treaty framework. Of course, individual organizations can and do include information about PGRFA accessions in their collections that are available under the Plant Treaty framework. Yet, to truly take advantage of the Plant Treaty framework as a whole, the information and related documentation concerning all of the materials globally available under the SMTA should be accessible from one source. 

In this paper, we analyze and compare the five most prominent data sources that have been developed to keep track of the PGRFA available under the Plant Treaty framework. We highlight the extent to which they provide identical, complementary and/or inconsistent information on the Plant Treaty framework PGRFA and the evident gaps where none of the five systems include this information, which we reasonably assume “must be out there”. We consider possible explanations for disparities in data records and make suggestions for future improvements to increase the transparency and lower transaction costs for people and organizations who want to share accession-level information, for those who want to access and use PGRFA under the Plant Treaty framework, and those who want to understand the full scope and function of the Plant Treaty framework. 

## 2. Materials and Methods

First, we analyzed Contracting Parties’ and natural or legal persons’ notification letters to the Governing Body/Secretariat from with respect to collections they announced as available under the Plant Treaty framework. Our analysis included all the notification letters posted from October 2006 (when the Secretariat invited the Contracting Parties to share information about collections in their country under the MLS or the same conditions) until 30 November 2021. We also analyzed data from Genesys, EURISCO, WIEWS) and GLIS. We compared the data obtained from all five data sources to ascertain the data consistency across the four platforms and the notifications on the Treaty Secretariat’s webpage. We note that, when comparing the total numbers of PGRFA accessions about which information is made available, it is important to recall EURISCO’s geographical focus, which only includes information on germplasm materials/accessions reported by European region countries that are eligible for membership of the European Cooperative Programme for Plant Genetic Resources (ECPGR). Furthermore, the presence of duplicate accessions in base and active collections should be considered when interpreting the results.

The five data sources compared in this paper were originally developed independently and for different purposes; therefore, they include heterogenous information. In the following paragraphs, we provide a brief description of each data source.

- Notification letters: The Treaty Governing Body Secretariat developed a sample notification letter to be used by Contracting Parties and natural or legal persons (but not international organization that signed Article 15 agreements) to declare available collections they hold under the Plant Treaty framework. This sample notification letter is available in four languages (Arabic, English, French and Spanish) on the Treaty’s website [6] and includes the following fields: name of the Contracting Party/natural or legal person; name of the collection center; location; name of species; and URL address, with additional information about the collection, including, presumably, accession-level information [5]. Eight of the nine Governing Body sessions have adopted resolutions inviting Contracting Parties and natural or legal persons to share such information. The Secretariat posts these notifications on the Plant Treaty webpage entitled “Material available in the Multilateral System” [5], which is updated when it receives new notifications letters. The Secretariat also posts a summary of relevant information, which is drawn from those notification letters, including the name of the country concerned and the total number of accessions under the MLS. This webpage also includes links to the text of Article 15 agreements signed by international organizations. These agreements do not include information about the content of the collections concerned.
- EURISCO: In 2003, the European Cooperative Programme for Plant Genetic Resources (ECPGR) [9] launched EURISCO to share accession-level information (passport and phenotypic data) about plant genetic resources conserved in *ex situ* collections maintained in ECPGR member or associate member countries. A total of 2,056,337 accessions (as of 30 November 2021) of crop plants and their wild relatives preserved by 401 institutes in 43 countries are listed in EURISCO [10]. ECPGR member countries, under ECPGR guidance, have recommended the policy to make non-Annex I materials available under the same terms and conditions as Annex I materials. Thus, EURISCO enables the specification for each accession whether it is included in the Multilateral System or is available under the SMTA (if non-Annex I), while the holding institutes retain full ownership and control over their PGRFA collections and associated data [10]. EURISCO is one of the data providers to Genesys and WIEWS [11,12].
- Genesys: In 2008, Bioversity International (now, the Alliance of Bioversity International and CIAT), on behalf of the System-wide Genetic Resources Programme of the CGIAR, the Global Crop Diversity Trust (GCDT), and the Secretariat of the Plant Treaty, launched Genesys as a collaborative project [11,13] to collate and share PGRFA accession-level information from sources worldwide, including Article 15 collections hosted by international organizations, national collections, and others. Since 2012, Genesys has been managed by the GCDT. Passport, characterization and evaluation data and images of 4,137,788 germplasm accessions are available through Genesys as of 30 November 2021 [11]. Networks, institutions and international, national and regional genebanks can contribute data to Genesys by signing a data provider’s agreement, while retaining full ownership and control over the data of their PGRFA collections. The GCDT is recognized as an essential element of the funding strategy of the Plant Treaty. Genesys includes data from over 500 collections around the world (including all the CGIAR Centers and the data from EURISCO) [11]. It does not include information about PGRFA in *in situ* or on-farm conditions.
- WIEWS: This database was originally launched in 1993, eight years before the Plant Treaty text was adopted [14], to facilitate information exchange and to periodically assess the state of conservation and use of the world’s PGRFA. In 2015, the Food and Agriculture Organization of the United Nations (FAO) Commission on Genetic Resources for Food and Agriculture (CGRFA) decided to integrate an information field into WIEWS [15,16] about whether or not accessions listed in WIEWS are included in the Multilateral System. The accession-level information available in WIEWS is limited to passport data; it does not include any PGRFA characterization and evaluation data and is thus less detailed than the accession-level information in Genesys. It does not include any information about PGRFA in *in situ* or on-farm conditions. As of August 2021, the WIEWS database contained information on 5,700,826 accessions, provided and updated by 114 member countries and 17 international/regional centers [13]. Through the WIEWS Reporting Tool, countries report to the FAO on the implementation of the Second Global Plan of Action for PGRFA (GPA II) every 3–5 years, and on a yearly basis, on Sustainable Development Goal (SDG) indicator 2.5.1a *Number of plant genetic resources for food and agriculture secured in either medium- or long-term conservation facilities.* Countries report accession-level information, with optional information about their designation under the MLS [13].
- GLIS: In 2017, as anticipated in Article 17 of the Plant Treaty (titled the “Global Information system on PGRFA”) [1], the Plant Treaty Secretariat launched GLIS [17]. The objective of the GLIS is to “facilitate the exchange of information, based on existing information systems, on scientific, technical and environmental matters related to plant genetic resources for food and agriculture”. Through the minting and use of GLIS digital object identifiers (GLIS-DOIs), it is possible to link accession-level information from multiple online information sources, including but not limited to Genesys, WIEWS and EURISCO [13,17]. To be included in or recognized by the GLIS, collection holders/managers must register each accession with a digital object identifier (DOI) through the DOI module [17]. The GLIS Scientific Advisory Committee and the Governing Body endorsed this option to better identify PGRFA and to improve how material can be referenced in third-party systems and in the scientific literature, with the expectation that setting such an international standard will facilitate data interoperability among different systems [13,17,18]. The DOI assignation is not limited to the genebank holdings, as it extends to any object, whether physical, digital, or abstract [19]. The GLIS portal DOI Registration module was launched in October 2017 [20]. The Governing Body welcomed this progress at its Seventh Session and requested that the Secretariat facilitate the assignment of DOIs on a voluntary basis through Resolution 5/2017 [21]. The use of DOIs remains voluntary. As of 30 November 2021, 1,187,393 DOIs had been recorded. DOIs can also be assigned to accessions that are not included in the MLS. At its Sixth Session, the Treaty Governing Body adopted Resolution 3/2015 containing a Program of Work for GLIS (PoW-GLIS) and its further development [22]. During its Ninth Session, the Governing Body reiterated the usefulness of the voluntary application of DOIs and the link between scientific publications and PGRFA data sets was encouraged. Furthermore, Contracting Parties, other governments and stakeholders have been invited to provide resources for the implementation of the PoW-GLIS, in particular to enhance the GLIS portal, review crop ontologies and support capacity development and technology transfer in developing countries in the next six years (2023–2028) [23]. EURISCO, Genesys and WIEWS include the GLIS-DOIs for accessions when they are included in the data submitted by the organizations hosting the materials [10,11,12].

The aforementioned four online databases have adopted the following data standards: (i) Multi-Crop Passport Descriptors (MCPDs) V.2.1 [24] are used to facilitate the exchange of germplasm passport information; (ii) GLIS-DOIs, as introduced above, are included in the MCPD standard [13,17]; and (iii) the WIEWS Institute Code [25], which is a unique alphanumeric identifier of the germplasm-holding organization, assigned by FAO after a registration procedure—this code is used worldwide in the exchange of information about germplasm maintained by national, regional and international institutes and organizations dealing with the conservation and sustainable use of PGRFA. Since taxonomy is naturally subject to change, data portals such as Genesys and EURISCO refer to the GRIN Taxonomy for Plants [26] and Mansfeld’s World Database of Agriculture and Horticultural Crops [27] to validate the correct scientific name of accessions, while WIEWS refers to Royal Botanic Gardens KEW’s Plants of the World Online [28]. During this analysis, the accession number, the accession taxon name and the WIEWS institute code are the main common indicators used. The accession number is an MCPD defined as a unique identifier that is assigned by the curator when an accession is entered into a genebank [25]. We reviewed the PGRFA taxonomy in the notification letters and validated it using the software R 4.3.2 and the package Taxonomic Standardization of Plant Species Names (Taxonstand) [29]. Based on “The Plant List” website [30], this package allows the automated standardization of taxonomic names and the removal of typing errors. The adoption of these standards provides for a strengthened interoperability among the systems [13].

## 3. Results of a Comparative Analysis of Five Data Information Sources about the Germplasm Available under the Plant Treaty Framework

**Figure 1:**
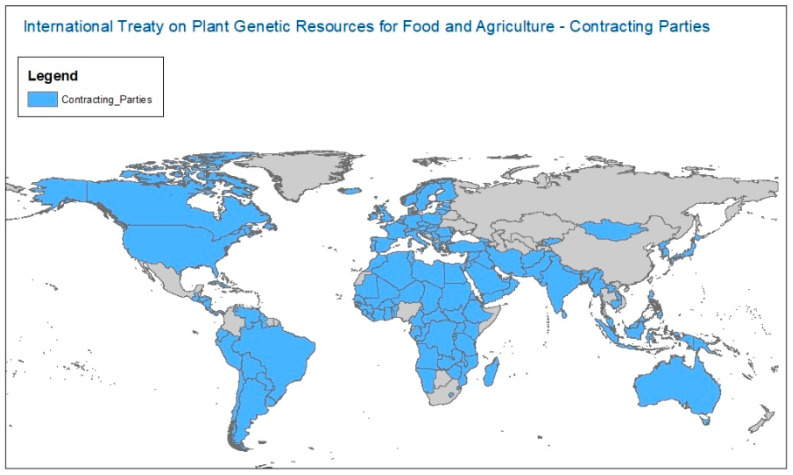
Plant Treaty Contracting Parties (as of November 2021).

After extensive discussions and acknowledging that data points evolve continuously over time, the study team set the cut-off point for this comparative analysis at 30 November 2021, at which time 148 countries (including the European Union as a Member Organization) had ratified or acceded to the Plant Treaty [31] (Figure 1) and 18 international organizations had signed Article 15 agreements to manage their in-trust collections under the Plant Treaty framework [6].

### 3.1. The Notification Letters on the Plant Treaty Website

As of 30 November 2021, the Secretariat of the Plant Treaty had received a total of 55 notification letters from 44 Contracting Parties and 6 notification letters from 6 natural or legal persons. There were no notification letters concerning PGRFA in the MLS from 70% of the Plant Treaty’s Contracting Parties. Some countries submitted more than one notification letter during this period. For instance, Belgium submitted five notifications, Japan three notifications and France, Italy, Lebanon, Poland and Spain two each (Table 1). 

The actual notification letters received by the Secretariat included very different levels of information, whether directly in the text of the letter or via a weblink. 

**Figure 2:**
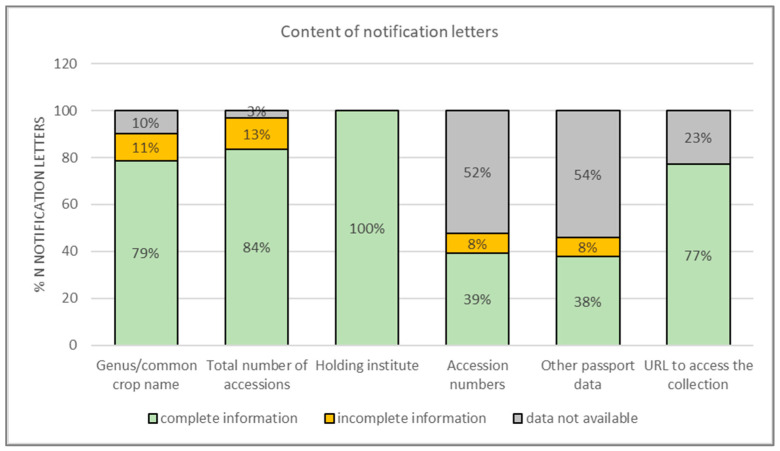
Percentage of notification letters that included information, directly or via a weblink, on the genus or common crop/forage name available under the MLS; total number of accessions; holding institutes; individual accession numbers; and other passport data. The graph also shows the percentage of notification letters that included URLs to access detailed information about the PGRFA. “Incomplete information” stands for notification letters that included information but not for all the PGRFA described in the notification letters.

Most of the letters provided information on the genus or the common name of the crop and forages available, the number of accessions, and the organization hosting those collections. Approximately 40% of the notification letters included individual accession numbers and passport information concerning those accessions (e.g., collecting mission identifiers, geographical coordinates, breeding institute, biological status, donor, type of germplasm storage, if it is an in vitro collection, a field genebank collection, a short-, medium- or long-term seed collection) (Figure 2).

Seventy-seven percent of the notification letters included links to websites where more information about the accessions concerned is curated and available (Figure 2). However, on the 30 November 2021, more than one-third of the URL links were broken. Finally, the notification letters from Austria and Armenia were not available on the Treaty’s website, although they were mentioned in the Secretariat’s summaries.

### 3.2. Comparison across the Five Data Sources regarding PGRFA from Plant Treaty Contracting Parties

Each of the data sources reported different total numbers of PGRFA as available under the Plant Treaty framework, with more than 400% differences between the WIEWS, which reported 947,800 PGRFA accessions as available, and GLIS, with information on 211,282 accessions (Table 2). Genesys, EURISCO and the notification letters fell in between, with 528,019, 424,176 and more than 643,356 PGRFA, respectively (Table 2).

Even when only considering the notification letters, there was a discrepancy in the total number of accessions under the MLS if we compared what was reported within the summaries and made available on the Plant Treaty’s website and the notification letters (text or URL) (Table 2). According to the Treaty Secretariat, these discrepancies are related to the link or reference that the notifier provided to the Secretariat, indicating that the updated figures can be found on the institute/genebank website. The Secretariat reported figures that stemmed from the national inventory, which differed from the data initially reported in the notification letter. Moreover, for periodic reports, the Secretariat used a combination of data sources, selecting the most up-to-date ones on a case-by-case basis. This made the reporting for each period of two years very complex. 

Regarding the number of countries related to holding institutes, the WIEWS includes PGRFA information held by 69 countries. The GLIS includes information for 50 countries, while Genesys and the notification letters for 34 and 44 countries, respectively (Table 2).

There was no information about the PGRFA available in the MLS from 79 Plant Treaty Contracting Parties held in the WIEWS, while there are 113 entries for Plant Treaty Contracting Parties in Genesys, 98 in the GLIS, and 106 through the notification letters. Finally, there are 57 Plant Treaty Contracting Parties for which no information was available on any of the five data sources (Table 2).

Even when the different data sources held data on PGRFA from the same countries, they were often radically different with respect to the number of accessions (Table 2). Comparing the holding institutes from the five data sources, when applicable, 69% of cases showed different data providers. For example, KEN015 and KEN122 are the holding institutes under the Plant Treaty framework for Kenya according to the notification letters. However, KEN212 is the holding institute according to Genesys and WIEWS, whereas no accession is reported by the GLIS. Even if KEN015 is kept for historical purposes only and has been invalidated and replaced by KEN212, the remaining discrepancy among the holding institutes largely explains the difference found in the number of accessions when exploring the different data sources.

Germany, Canada and Poland were the top-three countries regarding the number of accessions under the MLS notified on the Plant Treaty website (see Table 3). However, Germany, Australia and Czech Republic were the top-three countries according to Genesys. According to the WIEWS, Germany held first place, followed by Canada and Australia. In the GLIS, Germany, Japan and Italy were the countries that had the highest number of accessions under the Plant Treaty framework. Finally, it is interesting to note how the USDA, which held a big collection of materials under the MLS, did not appear when exploring Genesys and WIEWS and applying the search filter “included in MLS”.

**Figure 3:**
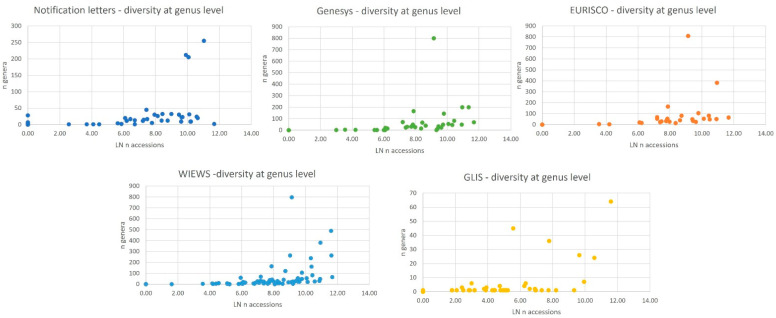
Number of genera corresponding to the accessions reported by the Contracting Parties under the Plant Treaty framework, according to the five data sources. LN stands for natural logarithm.

As a rough proxy for the genetic diversity of materials that Contracting Parties, natural or legal persons, and international institutions are making available through the Plant Treaty framework, we can refer to the number of different genera reported in the notification letters or included in the four online databases. Analyzing the number of different genera gives us an idea of the diversity of the accessions under the Plant Treaty framework (Table 2 and Figure 3). Only a few notifications letters referred to a single genus, for example, the letters submitted by Bhutan, Egypt, the Philippines, Uganda and Uruguay that notified, respectively, *Oryza*, *Citrus*, *Oryza*, *Phaseolus* and *Solanum*. Most of the notification letters referred to accessions belonging to 50 different genera or less. The situation was similar in Genesys, which, however, listed different countries as including PGRFA of a single genus, namely Brazil, Denmark, Finland, Kenya, Norway and Zambia. The WIEWS reported only three countries (Brazil, Greece and Uzbekistan) making available PGRFA of a single genus under the Plant Treaty framework (*Oryza*, *Citrus* and *Malus*, respectively). The notification letter with the highest number of genera and species was submitted by Poland, with accessions from 255 different genera; whereas Belgium, according to Genesys, EURISCO and WIEWS held the most diverse accessions included in the MLS or under the same conditions, belonging to about 800 different genera!

### 3.3. Comparison across Five Data Sources regarding PGRFA from Natural or Legal Persons

Information concerning the total PGRFA accessions (of different genera) available from natural or legal persons under the Plant Treaty framework, as reported in the five data sources consulted, in addition to the notification letters, is summarized in Table 4. Very little information was being made directly available from natural or legal persons, or explicitly in the names of natural or legal persons. Only three of the five data sources reported information received from them: notification letters, Genesys and EURISCO.

### 3.4. Comparison across Genesys, WIEWS and GLIS regarding PGRFA from Article 15 Organizations

The international organizations that signed Article 15 agreements were not included in the original invitation sent out by the Secretariat in 2006, which asked signatories to fill out the sample notification letter available online. None of the international organizations had ever submitted a notification letter, except for the Centre for Pacific Crops and Trees (CePaCT)—Pacific Community—(SPC). In most other instances, the other Article 15 organizations had used one or more of the four online databases described above to report on the accessions they maintained that were available under the Plant Treaty framework. 

The total number of accessions and the number of different genera conserved and managed by the Article 15 organizations and under the Plant Treaty framework are summarized in Table 5. 

**Figure 4:**
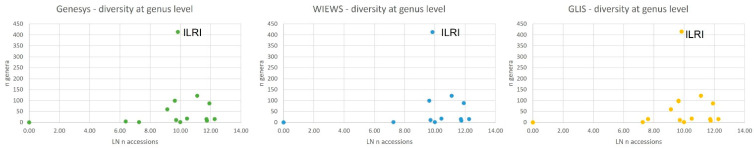
Genus-level diversity of the accessions under Article 15 and the Plant Treaty framework, according to Genesys, WIEWS and GLIS. LN stands for natural logarithm.

The data from the Article 15 organizations that reported accessions in Genesys, WIEWS and GLIS were very much aligned. Most of the differences were attributable to the different times and frequencies with which these databases were being updated. The most consistent data across all the information systems were from the CGIAR Centers and CATIE. Limited information was available on the other Article 15 organizations. Concerning the number of genera (Figure 4), ILRI had by far the highest number of genera and relatively few accessions per genus (i.e., 45) compared with, for instance, ICARDA (with an average of about 1706 accessions per genus), CIMMYT (with an average of about 14,056 accessions per genus) and IRRI (with an average of about 15,905). 

## 4. Discussion

### 4.1. PGRFA Available under the Plant Treaty Framework

The WIEWS includes information about the largest numbers of genebank accessions under long- and medium-term storage from Contracting Parties that are under the Plant Treaty framework. While it is beyond the scope of this paper to delve into why this is the case, it is logical that some contributing factors are as follows: WIEWS is the creation of FAO; it is an integral part of an FAO-endorsed strategy and infrastructure for monitoring and reporting on the Global Plan of Action for PGRFA. Many countries receive assistance from the FAO, or from other agencies supported by the FAO, to provide their periodic national reports to the WIEWS. It is a recognized responsibility of the national focal points for the FAO CGRFA to coordinate the development and submission of these reports. However, as noted above, the WIEWS generally includes fewer layers of accession-level information than Genesys, although it contains information that has been formally provided by the FAO member states on their PGRFA. Thus, it may be broader in coverage than Genesys, but it is not as deep.

By contrast, Genesys was not directly created by the FAO and is maintained and operated by and independent research organization, the GCDT. Therefore, Genesys does not have the same automatically recognized legitimacy/familiarity that the WIEWS enjoys, not being a creation of a United Nations agency such as the FAO. Genesys, therefore, needs to reach out to and engage with national institutes hosting PGRFA under the Plant Treaty framework to obtain their information. The number of national institutes entering into data-sharing agreements with Genesys is growing fairly rapidly. A growing percentage of these organizations are from developing countries. The GCDT provides financial support to the CGIAR genebanks and has historically worked very closely with these genebanks to establish and monitor performance targets. There is a very close connection between Genesys and the CGIAR-operated genebanks. Furthermore, given that the CGIAR was involved in developing and promoting Genesys in its initial phases, it is not surprising that these genebanks make information available through Genesys as a matter of course. 

The GLIS is a new online database that is still under development. It needs to be promoted and requires increased awareness raising and an advanced understanding of the technology it uses to face the challenges for which it was designed, for instance, minting DOIs. However, there is still an open debate within both the Governing Body of the Plant Treaty and the Scientific Advisory Committee on the Global Information System (SAC-GLIS) on the functions and the priorities for training support and collaboration. To date, many countries with extensive PGRFA collections have not undergone the process of assigning DOIs. It is not difficult to mint GLIS-DOIs, particularly considering the tools developed and assistance offered by the Plant Treaty Secretariat. However, it does require an active recognition of the GLIS, the value of DOIs, and the time necessary (which, again, is minimal) to initiate the process of assigning DOIs. The CGIAR has recognized the potential value of GLIS-DOIs for the global community as a means of aggregating, connecting and adding accession-level information to materials available under the Plant Treaty framework. 

Reporting individual accessions with their accession numbers and/or DOIs is an absolute precondition for meaningfully identifying and monitoring the individual accessions, and for the benefit-sharing arising from their use. Furthermore, as already mentioned, the GCDT has close ties with the Plant Treaty, and it also operates Genesys. Thus, through the coordination of the Genebank Platform and the operation of Genesys, the CGIAR Centers were among the first organizations to seek GLIS-DOIs for their international collections. They have been less proactive in minting GLIS-DOIs for breeding materials, although the ICRISAT has conducted very interesting pioneering work in this regard. An increasing number of organizations beyond the CGIAR are starting to mint GLIS-DOIs. As discussed below, this is a trend that should be encouraged in the coming years.

Increased cooperation among relevant institutions and initiatives to facilitate the exchange of information associated with PGRFA has been called for repeatedly by the Plant Treaty’s Governing Body and CGRFA. During its Seventeenth Regular Session, the CGRFA specifically requested that the FAO continue developing WIEWS and strengthening cooperation with the GLIS and Genesys to avoid the duplication of efforts and to reduce the reporting burdens [13,32]. As stated during the Tenth Session of the Intergovernmental Technical Working Group on Plant Genetic Resources for Food and Agriculture [13], this synergy would increase data volumes and quality, and further opportunities based on this collaboration will be explored, especially in relation to implementing SDG Target 2.5, the Plant Treaty and the Second GPA [13]. 

While the system of expecting Contracting Parties and natural or legal persons to send written notifications to the Secretariat to be posted verbatim on the Plant Treaty’s website was a necessary stop-gap measure at the time, it now seems out of date/obsolete, except for cases where countries face legal issues or technical problems in assigning DOIs. The creation, increased use, and utility of the four online databases discussed in this paper should render the notification letters redundant. However, it is recommended that the Plant Treaty webpage on the material under the MLS not be deleted. It could be replaced with summaries by the Plant Treaty Secretariat (possibly “mined” in “real time” from the databases) of the number of materials that Contracting Parties, natural or legal persons, and Article 15 organizations are making available under the Plant Treaty framework. This would be a way to recognize the efforts that organizations and individuals have made to share information and could also possibly create an incentive for the more proactive use of those online databases by Contracting Parties, natural or legal persons, and Article 15 organizations. 

A combination of technical and political factors appears to contribute to some Contracting Parties not making available any (or more) information about materials under the Plant Treaty framework through any of those online data sources. Technical factors include the poor state of national collections, uncertain levels of viability of reproductive materials and undeveloped information systems. One important political factor is that some would-be providers are reluctant to provide facilitated access to PGRFA when they are still not content with the level of monetary benefit-sharing that is being realized through that same system. In 2013, the Governing Body launched a process to enhance the functioning of the MLS, including developing a new formula for increasing payments from PGRFA users to the BSF. Unfortunately, these negotiations were suspended in 2019, by the Eighth Session of the Governing Body, resulting in outstanding tensions over monetary benefit-sharing not being eased. The negotiations were recently relaunched by a decision of the Ninth Session of the Governing Body in September 2022. This negotiation relaunch and re-examination of the MLS’s functioning provide an excellent opportunity to introduce systems, standards, incentives and possibly new requirements to share information about materials that are available under the Plant Treaty framework.

In general, at the time of this analysis, very little information and few accessions were the subject of notification letters from natural or legal persons or were attributed to natural or legal persons in the four online databases. A contributing factor could be that many Contracting Parties are not actively following up on their commitment under Article 11.3 of the Plant Treaty to develop policy measures to encourage natural or legal persons to make materials available. Another factor appears to be that natural or legal persons make materials available through national or international genebanks and therefore are not recognized as the parties making such materials available in the notification letters or databases.

From our experience, one of the most limiting factors in assessing the data quality of the various online data sources is the very irregular and non-synchronized updating times or intervals. A more timely and standardized updating effort seems to be the only solution. However, many of the data differences we have highlighted in this paper cannot be solely attributed to timing differences in terms of database updates. It also appears that the reporting process through national focal points is not always effective. Some institutes upload data directly, without national coordination or bypassing the designated channels. Therefore, the national focal points of those countries that do not have an integrated single genebank or a unified inventory should be informed by the notifying genebank and take necessary actions. Sometimes, the same accessions are counted twice when they are in both the active and base collections of the same organizations. Another aspect to take into account when considering “what materials are available” is the fact that some of the materials—we do not know how many—being conserved by different organizations are duplicates. Hence, some materials may be “counted” several times when considering global totals. It would be useful for stakeholders worldwide to coordinate efforts to identify duplicates in order to lower the overall costs of conservation.

The focus of our discussion until now has been on sharing information about PGRFA that are available under the Plant Treaty framework. Of course, the initial focus of such efforts is to obtain information loaded into and available through some combination of the information systems discussed. However, it should also be considered that some materials will cease to be available from some providers as a result of materials being lost, diseased, or found to be redundant as genebanks increasingly make use of genomic characterization data to analyze the structure of their collections. In such cases, Contracting Parties, international organizations and others will need to be able to update the information they have shared to indicate if a particular accession is no longer conserved or being made available by them. 

### 4.2. Technical Constraints Encountered When Analyzing Notification Letters and Online Databases 

Missing data, unclear data, typos in taxonomic names and out-of-date taxonomies affect, to different degrees, the quality of the information notified to the Plant Treaty. It is worth noting that only a few notification letters provide complete accession-level information. The sample notification letter available on the Plant Treaty’s webpage is often not filled out for all the elements. Thus, the accession-level information that is made available through the notification letters (including through genebank websites for which links are provided in the notification letters) is highly variable across collections, countries and natural or legal persons. These factors make it difficult to analyze and to compare data. Indeed, the accessions are submitted at the genus or species level, and they are listed with either their unique accession number or just as the total number of accessions for each crop or species. The use of common crop names instead of scientific names requires data processing for standardization and analysis.

Other technical issues encountered during this analysis concern limitations in the downloading process for information from online sources and in querying data through the genebank/institute websites and/or through each of the four web portals on PGRFA. For instance, Genesys and the Germplasm Resources Information Network (GRIN) place a limit on the number of records that can be downloaded; EURISCO does not have the facility to search by WIEWS institution code. The WIEWS and GLIS do not facilitate access to/exploration of the accessions online without downloading data. Furthermore, in more than one-third of notification letters that provide weblinks to access germplasm collections, there are broken URLs. Maintaining functional web links is certainly an additional and specific responsibility of the Contracting Parties, international centers and natural or legal persons, and this should be clearly pointed out by the Treaty to the countries/holders when making materials available under the Plant Treaty framework.

Finally, the discrepancy between the summaries written by the Plant Treaty Secretariat and posted on their webpage and the information retrievable through the actual notification letters is mainly due to genebank websites’ broken URLs provided in the same notification letters or by limitations in downloading and querying data, which affect some of those websites. Rectifying these shortcomings is the responsibility of the notifying country or genebank and the errors should be avoided or corrected upon request from the responsible person at the Secretariat.

## 5. Conclusions and Recommendations

At present, it is difficult to easily ascertain the full range of PGRFA that are included under the Plant Treaty framework. There are very useful information systems that provide accession-level information, but given their different original objectives, histories, institutional affiliations and levels of buy-in from organizations hosting PGRFA, they provide very different overall information about the amount and sources of PGRFA that are available under the Plant Treaty framework. While it is understandable how this situation evolved—partly as a result of the Plant Treaty not including a legal obligation for Contracting Parties, international organizations and others to provide such information—it would be a gamechanger to have a harmonized, frequently updated system that PGRFA users and researchers interested in the operation of the MLS could use to easily identify what PGRFA is available under the Plant Treaty framework.

In theory, each of the online databases analyzed in this study, with the exception of the EURISCO (as it has a specific regional coverage), has the potential to fulfil this key role in a more harmonized system. However, in our view, it makes most sense to promote the GLIS as the “go to” source for such information, primarily because of its potential capacity to integrate and link accession level information from multiple online information systems, including Genesys, WIEWS, EURISCO and other specialized online databases. The GLIS does not duplicate efforts; rather, it selectively gathers essential data for identification and linkage facilitation between the existing online systems, without replicating them. The WIEWS, Genesys and EURISCO would remain operational with their unique functionalities, objectives and established procedures. (Nevertheless, fostering a more harmonized system in terms of the data update timing and adopted standards would improve data quality within the whole system, thus avoiding data discrepancies.) A Treaty-operated database would be more authoritative than a database managed by any of the organizations. The GLIS can and should redirect users to these systems based on their needs and the particular strengths of those information systems. For example, the GLIS can link to Genesys when characterization and evaluation data are particularly relevant for researchers and plant breeders and when requests for materials are also relayed to the genebanks; the GLIS can also link to the WIEWS’ cross-sectional and time-series data on PGRFA preserved in genebanks, including their state of conservation, as well as information on more than 19,000 related publications as of June 2023. In short, routine minting and use of GLIS-DOIs by holders of PGRFA would contribute, over time, to a much richer, systematically ordered body of information about PGRFA that are available under the Plant Treaty framework. 

We are not blindly putting our faith in the GLIS; we appreciate that there are challenges facing its universal adoption and use, and challenges in terms of its interoperability with other existing systems. To date, many Contracting Parties have not shared any information, through any means, concerning PGRFA within their boundaries that is available under the Plant Treaty framework. Furthermore, experience over the last five years has demonstrated that there are significant barriers to entry for using the GLIS by Contracting Parties, international organizations and natural or legal persons who are willing and ready to share such information. These barriers arise from technical and internal legal constraints. At the fifth meeting of the SAC-GLIS, held in Rome on 8–9 May 2023, some national genebanks indicated that they need capacity-building to clean and upload the data into the GLIS as well as into the other online databases. It appears that many potential users are still not fully aware of the rationale, structure and function of the GLIS and how to use it, including how to mint and use GLIS-DOIs. 

The decision of the Ninth Session of the Plant Treaty Governing Body, in September 2022, to relaunch negotiations to improve the MLS provides an opportunity to adopt a new package of measures to address these challenges. One component of this package is increased monetary benefit-sharing. Increased benefit-sharing is clearly a prerequisite for many Contracting Parties to be willing to confirm what PGRFA within their borders are available through the MLS. In addition to promoting increased benefit-sharing, the revised package of measures could also include incentives to share information about materials that are available under the Plant Treaty framework by minting GLIS-DOIs. These incentives would need to be accompanied by increased resources for raising awareness about the GLIS and building the capacity of PGRFA holders and other stakeholders to use it.

The system of receiving notification letters from Contracting Parties is now obsolete, given the existence and enhanced functionality of the online databases. Therefore, the Plant Treaty’s website dedicated to the notification letters could be usefully replaced by a dashboard with real-time data visualization, summary statistics and useful resources derived from this potentially harmonized system.

## Figures and Tables

**Table 1:** Number of notification letters submitted to the Plant Treaty by Contracting Parties and natural or legal persons as of 30 November 2021.

Contracting Party	Number of Notifications	Contracting Party	Number of Notifications
Armenia	1	Madagascar	1
Australia	1	Malawi	1
Austria	1	Morocco	1
Belgium	5	Namibia	1
Bhutan	1	Nepal	1
Brazil	1	Netherlands	1
Burkina Faso	1	Philippine	1
Canada	1	Poland	2
Costa Rica	1	Portugal	1
Croatia	1	Romania	1
Cyprus	1	Rwanda	1
Czech Republic	1	Senegal	1
Egypt	1	Spain	2
Estonia	1	Sudan	1
France	2	Sweden	1
Germany	1	Switzerland	1
India	1	Tanzania	1
Italy	2	Uganda	1
Japan	3	United Kingdom	1
Jordan	1	Uruguay	1
Kenya	1	United States of America	1
Lebanon	2	Zambia	1
**Natural or Legal Person**	**Number of** **Notifications**	**Natural or Legal** **Person**	**Number of** **Notifications**
Association of Communities in the Potato Park	1	Costa Rica—Universidad de Costa Rica	1
India—Peermade Development Society	1	France—Association Française des Semences de céréales à paille et autres espèces Autogames (AFSA); the National Institute for Agricultural Research of France (INRA)	1
Kenya—Maseno University	1	France—Association pour l’Etude et l’Amélioration du Maïs (Pro-Maïs); the National Institute for Agricultural Research of France (INRA)	1

**Table 2:** Overall number of accessions and genera notified under the Plant Treaty framework by Contracting Parties. The column “Secretariat’s summary” refers to the data extracted from the notification letters by the Plant Treaty Secretariat and made available on the Plant Treaty’s website, where the notification letters can be downloaded. NA stands for “not available”. > stands for “more than” and applies when it was not possible to identify the full number of accessions or genera but it is known that the total number is greater than that actual reported in table below. ≈ stands for “about” and applies to accessions with “unidentified” genera. Status of data used: as available on 30 November 2021.

	Notification Letters			Genesys		EURISCO		WIEWS		GLIS	
	Secretariat’s Summary	Text of the Letter or URL									
Country	No. Accessions	No. Accessions	No. Genera	No. Accessions	No. Genera	No. Accessions	No. Genera	No. Accessions	No. Genera	No. Accessions	No. Genera
Afghanistan	0	0	0	0	0	0	0	953	13	0	0
Albania	0	0	0	2286	31	2343	32	2299	31	0	0
Armenia	1640	NA	NA	2504	48	2545	53	1088	27	0	0
Australia	NA	>20,000	212	84,476	>200	0	0	109,526	≈489	0	0
Austria	5487	NA	NA	5607	40	5607	40	5607	40	18	1
Azerbaijan	0	0	0	0	0	0	0	8386	263	0	0
Bangladesh	0	0	0	0	0	0	0	9917	24	0	0
Belarus	0	0	0	0	0	0	0	0	0	6	1
Belgium	>149	1773	>17	9318	>800	9318	>809	9309	≈797	138	1
Bosnia and Herzegovina	0	0	0	0	0	0	0	0	0	6	1
Bhutan	60	60	1	0	0	0	0	0	0	0	0
Brazil	2377	2377	5	11,121	1	0	0	3184	1	11,216	1
Bulgaria	0	0	0	67	2	67	2	67	2	43	2
Burkina Faso	16,479	>16,179	23	0	0	0	0	0	0	0	0
Burundi	0	0	0	0	0	0	0	0	0	188	1
Canada	100,500	23,473	>205	0	0	0	0	111,156	264	0	0
Chile	0	0	0	0	0	0	0	0	0	6	1
Costa Rica	NA	Not found	7	0	0	0	0	164	3	0	0
Croatia	387	442	20	442	20	442	20	442	20	12	1
Cyprus	485	485	11	504	15	504	15	504	15	6	1
Czech Republic	32,616	14,863	9	56,178	>200	56,716	381	56,314	381	12	1
Denmark	0	0	0	431	1	0	0	423	4	24	1
Ecuador	0	0	0	0	0	0	0	13,546	55	50	3
Egypt	40	40	1	0	0	0	0	10,998	23	0	0
Eritrea	0	0	0	0	0	0	0	1205	17	0	0
Estonia	2496	3407	26	2897	26	2897	26	2897	26	24	1
Ethiopia	0	0	0	0	0	0	0	52,657	29	0	0
Finland	0	0	0	400	1	0	0	0	0	120	1
France	1341	1349	11	4235	14	4235	14	4235	14	1020	1
Germany	108,675	117,110	>2	117,404	68	117,257	64	117,256	66	108,203	64
Ghana	0	0	0	0	0	0	0	163	8	8	1
Greece	0	0	0	0	0	0	0	5	1	24	1
Guinea	0	0	0	0	0	0	0	96	9	0	0
Guyana	0	0	0	0	0	0	0	81	5	0	0
Honduras	0	0	0	0	0	0	0	64	6	0	0
Hungary	0	0	0	2617	165	2617	164	2617	165	168	1
India	26,523	26,523	9	0	0	0	0	0	0	1513	1
Indonesia	0	0	0	0	0	0	0	332	2	1058	1
Italy	46,788	46,788	Not found	16,821	48	12,075	49	17,482	48	20,720	7
Japan	40,000	38,960	24	0	0	0	0	38,959	25	38,959	24
Jordan	1885	>1885	Not found	0	0	0	0	2335	37	2389	36
Kenya	12,873	12,873	30	225	1	0	0	25,054	20	0	0
Kyrgyzstan	0	0	0	0	0	0	0	1382	27	0	0
Latvia	0	0	0	1751	29	1751	29	1308	29	0	0
Lebanon	274	1676	45	0	0	0	0	380	≈59	261	45
Madagascar	7999	>7999	32	0	0	0	0	7563	17	0	0
Malawi	1419	1419	15	0	0	0	0	2702	≈42	20	6
Malaysia	0	0	0	0	0	0	0	9998	4	721	2
Mali	0	0	0	0	0	0	0	2078	5	0	0
Mongolia	0	0	0	0	0	0	0	1197	12	0	0
Montenegro	0	0	0	35	4	35	4	35	4	0	0
Morocco	NA	>352	>2	0	0	0	0	0	0	0	0
Namibia	1441	Not found	Not found	0	0	0	0	0	0	0	0
Nepal	614	614	17	0	0	0	0	0	0	0	0
The Netherlands	15,218	>15,226	>22	15,029	23	15,179	25	15,042	23	15,440	26
Niger	0	0	0	0	0	0	0	3876	10	0	0
Norway	0	0	0	20	1	0	0	2059	13	24	1
Pakistan	0	0	0	0	0	0	0	30,643	≈239	0	0
Panama	0	0	0	0	0	0	0	391	6	0	0
Papua New Guinea	0	0	0	0	0	0	0	1615	6	0	0
Peru	0	0	0	0	0	0	0	5258	4	17	1
The Philippines	811	811	1	0	0	0	0	4271	9	3675	1
Poland	61,627	61,627	255	54,649	48	55,089	50	54,619	48	519	4
Portugal	813	813	13	0	0	0	0	31,884	161	2292	1
Qatar	0	0	0	0	0	0	0	0	0	1	1
Republic of Ireland	0	0	0	1601	23	1601	23	1601	23	24	1
Republic of Lithuania	0	0	0	1326	70	1326	68	1326	68	0	0
Romania	6363	2790	30	4531	66	6180	82	6251	121	150	1
Russian Federation	0	0	0	0	0	0	0	0	0	6	1
Rwanda	NA	Not found	>28	0	0	0	0	0	0	0	0
Senegal	49	Not found	3	0	0	0	0	898	5	0	0
Serbia	0	0	0	0	0	0	0	0	0	114	4
Slovakia	0	0	0	12,629	34	12,629	34	12,629	34	0	0
Slovenia	0	0	0	1332	54	1332	54	0	0	48	1
Spain	41,521	41,521	>20	23,503	54	24,877	54	23,517	54	1000	2
Sudan	6351	6351	12	11,816	11	0	0	9002	19	0	0
Sweden	24,713	24,713	31	30,292	45	35,934	48	30,523	NA	84	1
Switzerland	25,507	25,507	Not found	33,965	82	33,965	82	33,868	82	72	1
Tajikistan	0	0	0	0	0	0	0	3782	28	0	0
The former Yugoslav Republic of Macedonia	0	0	0	0	0	0	0	0	0	6	1
Togo	0	0	0	0	0	0	0	845	7	0	0
Tunisia	0	0	0	0	0	0	0	13,780	23	0	0
Turkey	0	0	0	0	0	0	0	0	0	156	1
Uganda	760	87	1	0	0	0	0	2236	12	566	6
United Kingdom	42,722	>27,390	9	17,746	144	17,655	105	17,485	105	138	1
United Republic of Tanzania	NA	277	4	0	0	0	0	0	0	0	0
United States of America	NA	4600	32	0	0	0	0	0	0	6	1
Uruguay	13	13	1	0	0	0	0	0	0	0	0
Uzbekistan	0	0	0	0	0	0	0	189	1	0	0
Zambia	4340	>4340	>12	261	1	0	0	4246	15	11	3
TOT Accessions	>643,356	>556,713		528,019		424,176		947,800		211,282	

**Table 3:** Top-three countries regarding the number of accessions under the MLS notified by Contracting Parties according to Plant Treaty website, Genesys, WIEWS and GLIS, based on data reported in Table 2.

	Notification Letters				Genesys		WIEWS		GLIS	
	Secretariat’s Summary		Text of the Letter or URL							
#	Country	No. Accessions	Country	No. Accessions	Country	No. Accessions	Country	No. Accessions	Country	No. Accessions
1.	Germany	108,675	Germany	117,110	Germany	117,404	Germany	117,256	Germany	108,203
2.	Canada	100,500	Poland	61,627	Australia	84,476	Canada	111,156	Japan	38,959
3.	Poland	61,627	Italy	46,788	Czech Republic	56,178	Australia	109,526	Italy	20,720

**Table 4:** Overall number of accessions and genera notified under the Plant Treaty framework by natural or legal persons. The column “Secretariat’s summary” refers to the data extracted from the notification letters and made available on the Plant Treaty’s website, where the notification letters are downloadable. NA stands for not available. Status of data used: as available on 30 November 2021.

Natural or Legal Person	Notification Letters			Genesys		EURISCO		WIEWS		GLIS	
	Secretariat’s Summary	Text of the Letter or URL									
	No. Accessions	No. Accessions	No. Genera	No. Accessions	No. Genera	No. Accessions	No. Genera	No. Accessions	No. Genera	No. Accessions	No. Genera
Assoc. of Comm. Potato Park	NA	Not found		0	0	0	0	0	0	0	0
India—Peermade Development Society	7	7	4	0	0	0	0	0	0	0	0
Kenya—Maseno University	12	12	1	0	0	0	0	0	0	0	0
Costa Rica—Universidad de Costa Rica	NA	128	1	0	0	0	0	0	0	0	0
France—AFSA and INRA	1784	1784	1	2983	4	2983	4	0	0	0	0
France—Pro-Maïs INRA	533	533	1	0	0	0	0	0	0	0	0
TOT Accessions	>2336	>2464		2983		2983		0		0	

**Table 5:** Overall number of accessions and genera under the Plant Treaty framework, conserved and managed by Article 15 organizations. Status of the data on 30 November 2021.

International Institute	Genesys		WIEWS		GLIS	
	No. Accessions	No. Genera	No. Accessions	No. Genera	No. Accessions	No. Genera
Africa Rice	21,812	1	21,812	1	21,812	1
Bioversity International	1424	1	1424	1	1424	1
CIAT	66,599	122	66,599	122	66,749	122
CIMMYT	210,851	15	210,851	15	211,315	15
CIP	16,477	11	16,477	11	16,578	11
ICARDA	148,419	≈87	148,419	88	147,135	87
ICRAF	15,157	99	15,157	99	15,156	99
ICRISAT	123,425	15	123,051	15	122,870	15
IITA	33,758	17	33,758	17	36,156	17
ILRI	18,638	≈414	18,638	413	18,636	415
IRRI	127,240	8	127,241	8	127,240	8
Centre for Pacific Crops and Trees (CePaCT)—Pacific Community—(SPC)	592	4	0	0	2025	15
International Center for Biosaline Agriculture (ICBA)	15,141	99	0	0	15,141	97
International Cocoa Genebank	0	0	0	0	0	0
International Coconut Genebank for African and the Indian Ocean	0	0	0	0	0	0
International Coconut Genebank for the South Pacific	Not found	Not found	Not found	Not found	Not found	Not found
Mutant Germplasm Repository of the FAO/IAEA Joint Division	0	0	0	0	0	0
Tropical Agricultural Research and Higher Education Center (CATIE)	9275	60	9297	61	9190	60
TOT Accessions	808,808		792,724		811,427	
